# Peripheral Blood Leukocyte Subpopulation Changes in Reaction to an Acute Psychosocial Stressor as Compared to an Active Placebo-Stressor in Healthy Young Males: Mediating Effects of Major Stress-Reactive Endocrine Parameters

**DOI:** 10.3390/cells13231941

**Published:** 2024-11-22

**Authors:** Lisa-Marie Walther, Angelina Gideon, Christine Sauter, Marcel Leist, Petra H. Wirtz

**Affiliations:** 1 Biological Work and Health Psychology, University of Konstanz, 78457 Konstanz, Germany; lisa-marie.walther@uni-konstanz.de (L.-M.W.);; 2 Centre for the Advanced Study of Collective Behaviour, University of Konstanz, 78457 Konstanz, Germany; 3 In Vitro Toxicology and Biomedicine, Department of Biology, University of Konstanz, 78457 Konstanz, Germany

**Keywords:** psychosocial stress, TSST, placebo-TSST, immune cell redistribution, epinephrine, renin, stress hemoconcentration

## Abstract

Psychosocial stress has been proposed to induce a redistribution of immune cells, but a comparison with an active placebo-psychosocial stress control condition is lacking so far. We investigated immune cell redistribution due to psychosocial stress compared to that resulting from an active placebo-psychosocial stress but otherwise identical control condition. Moreover, we tested for mediating effects of endocrine parameters and blood volume changes. The final study sample comprised 64 healthy young men who underwent either a psychosocial stress condition (Trier Social Stress Test; TSST; *n* = 38) or an active placebo-psychosocial stress control condition (PlacTSST; *n* = 26). Immune cell counts and hemoglobin, epinephrine, norepinephrine, ACTH, renin, and aldosterone levels, as well as those of saliva cortisol, were determined before and up to 30 min after the TSST/PlacTSST. The TSST induced greater increases in total leukocyte, monocyte, and lymphocyte levels as compared to the PlacTSST (*p*’s ≤ 0.001), but in not granulocyte counts. Neutrophil granulocyte counts increased in reaction to both the TSST and PlacTSST (*p*’s ≤ 0.001), while eosinophil and basophil granulocyte counts did not. The psychosocial stress-induced increases in immune cell counts from baseline to peak (i.e., +1 min after TSST cessation) were independently mediated by parallel increases in epinephrine (ab’s ≤ −0.43; 95% CIs [LLs ≤ −0.66; ULs ≤ −0.09]). Subsequent decreases in immune cell counts from +1 min to +10 min after psychosocial stress cessation were mediated by parallel epinephrine, renin, and blood volume decreases (ab’s ≥ 0.17; 95% CIs [LLs ≥ 0.02; ULs ≥ 0.35]). Our findings indicate that psychosocial stress specifically induces immune cell count increases in most leukocyte subpopulations that are not secondary to the physical or cognitive demands of the stress task. Increases in the number of circulating neutrophil granulocytes, however, are not psychosocial stress-specific and even occur in situations with a low probability of threat or harm. Our findings point to a major role of epinephrine in mediating stress-induced immune cell count increases and of epinephrine, renin, and blood volume changes in mediating subsequent immune cell count decreases from +1 min to +10 min after psychosocial stress cessation.

## 1. Introduction

Acute stress elicits various physiological reactions that allow us to efficiently respond to threat (e.g., fight-or-flight) and to cope with potential consequences (e.g., injuries, tissue damages, inflammation, antigen or pathogen entry) [1,2,3,4,5,6]. Major stress-reactive endocrine systems comprise the sympathetic–adrenal–medullary (SAM) axis with the catecholamines epinephrine (EP) and norepinephrine (NEP) and the hypothalamus–pituitary–adrenal (HPA) axis with the circulating stress hormones adrenocorticotropic hormone (ACTH) and cortisol [7]. The activation of the SAM and HPA axes can lead to the activation of additional major physiological systems, including the immune system [8,9,10]. In particular, acute stress has been proposed to induce a redistribution of immune cells from storage organs into the circulation, increasing the capacity of the immune system to rapidly respond to impending challenges in case of injury, tissue damage, or antigen or pathogen entry [9,11]. As a consequence, immune surveillance and functioning is enhanced [11]. Notably, acute stress also induces a transient acute loss of blood volume (BV) into the extravascular space, resulting in the concentration of and the passive increase in larger (>69 kDa), non-diffusible blood constituents such as leukocytes [12]. Therefore, the effects of stress-induced hemoconcentration should be considered as a confounding factor when investigating immune cell redistribution in the context of stress [12,13]. Laboratory mental stressors can be categorized as mild (i.e., cognitive tasks), moderate (i.e., public speaking tasks), and strong (i.e., the combination of cognitive with public speaking tasks) mental stressors based on the stressors’ capacity to induce physiological stress reactions [14,15]. More precisely, meta-analytic findings revealed that cognitive tasks induce cortisol reactions of small, public speaking tasks of moderate, and the combination of cognitive and public speaking tasks of large effect sizes. Notably, as strong mental stressors combine social evaluative threat and uncontrollability, they are often referred to as psychosocial stressors. A frequently used psychosocial stressor is the Trier Social Stress Test (TSST) [16], which comprises a short introduction phase followed by a 3 min preparation phase, a 5 min mock job interview, and 5 min mental arithmetic task in front of an audience with video and audio recording.

So far, several studies have investigated the redistribution of immune cells (mostly assessed by absolute counts) in reaction to acute mental stress in healthy individuals. In reaction to acute mental stressors of all intensities, significant increases in the number of total leukocytes and neutrophil granulocytes and lymphocytes (the largest leukocyte subpopulations [17]) have been frequently observed during and up to 10 min after stress cessation in healthy individuals, both with and without control for stress hemoconcentration effects [18,19,20,21,22,23,24,25,26,27,28,29,30,31,32,33,34,35,36,37,38,39,40,41,42,43,44,45,46]. With respect to monocytes, studies that applied moderate or strong mental stressors and controlled for stress hemoconcentration effects consistently showed comparable increases in monocyte counts during and up to 10 min after stress cessation [31,37,39], whereas mild stress induction was not capable of inducing changes in monocyte counts [38]. Studies that did not control for hemoconcentration effects had mixed results, independent of the stressor intensity [18,19,20,21,23,25,26,30,31,33,34,35,36,37,38,43,44,45,47,48,49]. Results for basophil granulocytes were similar, with significant increases in basophil granulocyte counts following strong mental stress without [30,31] but not with control for stress hemoconcentration [31], and no changes in reaction to mild or moderate mental stress [39,47,48]. With respect to eosinophil granulocytes, the vast majority of studies did not observe changes in eosinophil granulocyte counts in reaction to acute mental stress, including mental stressors of strong intensity, without or with control for stress hemoconcentration effects [30,31,39,47,48,50]. Studies examining immune cell counts +10−30 min after stress cessation show that the increased immune cell counts return to baseline levels between +15 and +30 min after stress cessation, independent of stress hemoconcentration effects [23,31,35,38,39].

Despite the above-described body of evidence, important questions remain. First, regarding the comparison with a control condition, all hitherto published studies assessing immune cell count reactivity to acute mental stress compared immune cell count changes after stress either with a single non-stress baseline measurement (e.g., [21,31,36]) or with a non-stress resting control condition (e.g., [21,31,36]). Drawing conclusions about specific mental stress effects on immune cell counts is not possible based on these study designs. This would require an active placebo-stress control condition which is highly comparable to the mental stress condition with respect to physical and cognitive demands (e.g., standing, speaking, calculating) but which lacks the mental stress-inducing components of the mental stress condition [51,52]. Notably, the placebo-TSST (PlacTSST [53]) represents an active placebo psychosocial-stress control condition that is identical to the TSST except for its psychosocial stress-inducing components [51,52], i.e., uncontrollability and social evaluation [14]. Notably, although the PlacTSST does not induce psychosocial stress in terms of uncontrollability and social evaluation, it nevertheless may induce minor physical and/or mental stress (e.g., orthostatic effects), as indicated by the reported minor but significant activation of the SAM axis and the renin–angiotensin–aldosterone system (RAAS) in reaction to the PlacTSST [53,54]. Second, the mechanisms underlying the redistribution of immune cells following acute stress are not fully understood. (1) Immune cell count increases: in addition to the above-described effect of stress hemoconcentration [12,13,29], it has been proposed that the stress-induced release of the catecholamines NEP and EP mobilizes the rapid trafficking of leukocytes from storage organs into the blood stream [9]. Notably, immune cells are capable of expressing both alpha- and beta-adrenergic receptors [55]. Evidence supporting the proposed mediation by NEP and EP comes, on the one hand, from cross-sectional studies that report positive associations between stress-induced catecholamine increases and immediate immune cell count changes [19,31,46]. On the other hand, infusion studies in humans have revealed that the infusion or injection of either NEP [31,56,57,58,59,60] or EP [57,58,60,61,62,63,64] induced significant immediate increases in immune cell counts. Notably, the infusion of EP dose-dependently elicits not only EP increases but also the concomitant release of NEP, whereas NEP-infusion increases NEP level only [31,65]. Moreover, negative associations between rapid increases in total leukocyte counts and parallel changes in cortisol [44] point to potential immunosuppressive effects of cortisol on immediate immune cell count increases following stress. (2) Immune cell count decreases: the decreases in immune cell counts that follow rapid stress-induced increases, these decreases presumably result from immune cells trafficking out of the blood into target cells such as those in sides of wounding, tissue damage, antigen/pathogen entry, and homeostatic surveillance and homing pathways, or, if not needed elsewhere, back to the storage organs [9]. It has been suggested that these immune cell count decreases are driven by changes in EP, NEP, and cortisol levels [9]. While the role of EP in immune cell count recovery has not yet been investigated, one study has tested for associations between immune cell count recovery after stress and NEP and cortisol changes in humans [33]. There were no associations between NEP recovery and monocyte, lymphocyte, or granulocyte count recovery. Findings regarding cortisol point to differential associations with lymphocytes and total granulocytes, with a negative association between lymphocyte count recovery and cortisol changes and, at the same time, with a positive association between granulocyte count recovery and cortisol changes [33]. A comprehensive investigation of the mediating role of the SAM and HPA axes hormones (i.e., EP, NEP, ACTH, and cortisol), as well as of BV changes in the immune cell count reactivity, following acute mental stress is lacking so far. (3) The role of further stress-reactive systems: regarding stress-reactive endocrine systems beyond the SAM and HPA axes, associations with the RAAS [54,66] have not yet been investigated in the context of acute stress-induced immune cell redistribution.

Here, we set out to examine, for the first time, the specifically psychosocial stress-induced redistribution of immune cells. To this end, we compared immune cell count changes in response to an acute psychosocial stress condition to those in response to an active placebo-psychosocial stress control condition with a similar setting and tasks as in the psychosocial stress condition but without the psychosocial stress-inducing elements social evaluation and uncontrollability. This study design allows us to verify that the proposed effects of acute psychosocial stress on immune cell redistribution are not secondary to the physical or cognitive demands of the task and setting of the psychosocial stress condition but are specifically attributable to psychosocial stress. We expected higher increases in total leukocyte, monocyte, lymphocyte, and neutrophil granulocyte counts in reaction to acute psychosocial stress induced by the TSST as compared to the PlacTSST. Moreover, to further elucidate the underlying mechanisms, we investigated whether psychosocial stress-induced immune cell count increases from baseline to +1 min after TSST cessation as well as subsequent decreases from +1 min to +10 min after TSST cessation, i.e., immediate recovery, are mediated by endocrine stress axes including changes in the RAAS and/or BV. In line with the findings of Dhabhar et al. and their proposed model [9], we particularly expected increases in EP and NEP to mediate psychosocial stress-induced immediate increases in leukocyte subpopulations. Moreover, we hypothesized that decreases in EP and NEP observed between +1 and +10 min after stress cessation, as well as lasting cortisol increases during this time period, would mediate the decreases in leukocyte subpopulations observed between +1 and +10 min after TSST cessation.

## 2. Materials and Methods

### 2.1. Study Participants

This study is part of a larger project assessing psychobiological reactivity to acute psychosocial stress in healthy young men [54,66]. The project was formally approved by the Ethics Committee of the University of Konstanz, Germany and conducted in accordance with the Declaration of Helsinki principles. All participants provided written informed consent prior to participation and were financially compensated for their participation. To exclude potential confounding by sex differences in the number and function of immune cells and in the innate and adaptive immune response [67,68], as well as by the sex differences in the SAM and HPA axes reactivity to acute stress [69,70], we intentionally restricted participation to male participants. As previously described in detail [54,66], we recruited a total of 70 healthy, medication-free, non-smoking men between 18 and 30 years. Using a placebo-controlled single-blind between-subject design, participants were randomly assigned to either a psychosocial stress experimental condition (“TSST condition”, *n* = 40 TSST participants) or an active placebo-psychosocial stress control condition (“PlacTSST condition”, *n* = 30 PlacTSST participants) in an age-matched manner (±1 year). Six of the recruited 70 participants had to be excluded due to venous catheter occlusion and/or severe problems with blood sampling during up to 30 min after TSST/PlacTSST cessation. Our final sample for this part of the study comprised a total of 64 participants, with 38 TSST participants and 26 PlacTSST participants.

### 2.2. Study Design and Procedure

Participants abstained from strenuous physical activity and alcohol for 48 h and from any kind of physical activity or chocolate or caffeine consumption for 24 h prior to their study participation. Moreover, participants were asked to keep a normal sleeping pattern on the study day, i.e., to get up between 7 and 8 AM, and to avoid sour, caffeinated, or sweet drinks. Furthermore, participants were asked to abstain from vaccination for four weeks and from dental surgery for two weeks prior to study participation.

Participants arrived at the laboratory of the Biological Work and Health Psychology Group at the University of Konstanz at 11 AM, where they received a standardized meal comprising two bottles of Fresubin^®^ (Fresenius Kabi Germany GmbH, Bad Homburg, Germany, 200 mL and 300 kcal per bottle), one piece of crispbread, and water. Afterwards, body weight and height were assessed. About 30 min after arrival, a venous catheter (Vasofix^®^ Safety Braunule green/white G 18, Fa. B. Braun, Melsungen, Germany) was inserted into the nondominant arm. After a subsequent 50 min acclimatization phase, the first, i.e., baseline, blood sample (−1 min) was obtained. Then, the participants underwent either the TSST [16] or the PlacTSST [53] (see subsection titled Acute Psychosocial Stress Induction) as experimental manipulation. Immediately after cessation of the TSST or PlacTSST, a second blood sample was taken (+1 min). Further samples followed +10, +20, and +30 min after TSST/PlacTSST cessation, while participants remained seated in a quiet room. Resting blood pressure (BP) was assessed by sphygmomanometry (Omron, M6, Nufringen, Germany) twice: 10 min before catheter insertion and immediately before the first blood sampling. Mean arterial pressure (MAP) was calculated as 2/3 diastolic BP + 1/3 systolic BP.

### 2.3. Acute Psychosocial Stress Induction

To induce acute psychosocial stress, we applied the standard protocol of the TSST [16] that reliably elicits stress responses in various physiological parameters including immune cell counts [31,34]. The TSST comprises a short introduction phase followed by a 3 min preparation phase, a 5 min mock job interview, and a 5 min mental arithmetic task in front of an audience with video and audio recording. Except for the preparation phase in sitting position, participants are required to accomplish all other phases in standing position.

Participants in the active placebo-psychosocial stress control condition underwent the placebo version of the TSST [53], which is structured corresponding to the TSST but without its psychosocial stress-inducing aspects, i.e., uncontrollability and social-evaluative threat. It comprises a short introduction phase followed by a 3 min preparation phase, a 5 min free speech about a recent positive personal experience, and a 5 min simple mental arithmetic task but without audience and recording. Notably, in the induction phase, participants are explicitly informed that they were assigned to take part in the control condition of the study. The PlacTSST controls for non-psychosocial stress-induced activation by physical and/or cognitive demands of the TSST including potential orthostatic and posture effects.

### 2.4. Psychological Assessment

#### Chronic Stress

To control for participants’ chronic stress in our statistical analyses, given the proposed effects of chronic stress on resting immune cell counts as well as on immune cell redistribution to acute stress [4], we applied the 12-item Chronic Stress Screening Scale of the Trier Inventory for Chronic Stress (TICS-CSSS) [71]. The questionnaire comprises descriptions of situations and experiences of worries (4 items), work overload (4 items), lack of social recognition (2 items), excessive demands at work (1 item), and social overload (1 item). Using a 5-point rating scale ranging from 0 (never) to 4 (very often), participants are asked about the frequency with which they encountered these situations and experiences within the last three months. By adding up all items, a total score between 0 and 48 is obtained. Higher scores indicate greater levels of chronic stress. Internal consistency was found to be very good in a representative German sample (*n* = 2339) [72]. In our sample, Cronbach’s α for TICS-CSSS was 0.91. Data of 3 TSST participants and 1 PlacTSST participant were missing due to incompletion.

### 2.5. Biochemical Analyses

For biochemical analysis of blood parameters, venous blood was drawn into two EDTA-coated monovettes (Sarstedt, Numbrecht, Germany) at each sampling timepoint. In more detail, blood for determination of immune cell counts and hemoglobin concentrations was drawn into 2.7 mL EDTA-coated monovettes and immediately processed at room temperature. Blood for the determination of endocrine parameters from plasma was drawn into 9 mL EDTA-coated monovettes and immediately cooled on ice for a maximum of 10 min until further processing. Here, blood samples were centrifugated at 2000× *g* and 4 °C for 10 min and plasma was stored at −80 °C until analysis. Saliva samples were collected using salivettes (Sarstedt, Rommelsdorf, Germany), centrifugated at 2500 rpm at room temperature for 10 min (Megafuge 40 R, Heraeus, Thermo Fisher Scientific, Langenselbold, Germany), aliquoted, and stored at −20 °C until analysis.

#### 2.5.1. Peripheral Blood Leukocyte Subpopulation Counts

Total leukocyte counts in addition to subpopulation counts, i.e., monocyte, granulocyte (i.e., neutrophil, basophil, and eosinophil granulocyte), and lymphocyte counts, were determined immediately after blood sampling at five sampling timepoints (−1, +1, +10, +20, +30 min) by acquiring five-part differential immune cell counts using an automated hematology system (XN-350; Sysmex GmbH, Norderstedt, Germany). The XN-350 device directly loads a total of 25 μL whole blood per measurement and was regularly maintained according to the manufacturer’s specifications, including a weekly cleaning sequence. Of a total of 320 samples (64 participants, 5 samples per participant), 4 samples within the recovery period after the peak, i.e., +10, +20, or +30 min after TSST/PlacTSST cessation, were missing. To prevent listwise exclusion of these participants with single missing measurements and to maximize statistical power, we replaced these missing values with the mean of the respective preceding and subsequent measurement, or, in case of missing samples at +30 min, by the respective preceding measurement. Immune cell counts were corrected for BV changes following recommended methods for whole blood cells [13] to account for stress effects on hemoconcentration [73] (see below). Statistical analyses were performed with both original and corrected immune cell counts.

#### 2.5.2. Blood Volume Changes

In addition to immune cell counts, we obtained hemoglobin (Hb) concentrations for all sampling timepoints. We controlled stress hemoconcentration effects in our analyses and additionally tested for mediation of stress-induced immune cell count changes by BV changes [13]. For stress hemoconcentration-corrected immune cell counts, we first calculated the relative changes (%) in immune cell counts or circulating biomarkers (BMs), respectively, using the formula ∆BM = (BM_post/_BM_pre_) × (Hb_pre/_HB_post_) − 1 [13]. Based on the determined relative changes in immune cell counts and absolute immune cell counts at baseline assessment, we then calculated absolute changes (10^3^/μL) in immune cell counts. BV changes for mediation testing were calculated using the formula ∆BV = 100 × (Hb_before_/Hb_after_) − 100 [12,13].

#### 2.5.3. Endocrine Parameters of Major Stress Axes

Sympathetic adrenal medulla (SAM) axis: To assess endocrine parameters of the SAM axis, the catecholamines EP and NEP were determined from plasma at 3 blood sampling timepoints (−1, +1, +10 min) by high-pressure liquid chromatography using electrochemical detection after liquid–liquid extraction [74] in the Laboratory for Stress Monitoring (LSM, Hardegsen, Germany). Intra-assay coefficients of variance (CVs) for EP and NEP were 3.0% and 3.7%, respectively. Detection limit was 8 pg/mL. Catecholamine data for 5 TSST participants and 3 PlacTSST participants were missing due to technical problems.

Hypothalamus–pituitary–adrenal (HPA) axis: As endocrine parameters of the HPA axis, we determined plasma ACTH and free salivary cortisol (CORT) at 5 sampling timepoints (−1, +1, +10, +20, +30 min). We used commercial enzyme-linked immunosorbent assay (ELISAs) according to the manufacturer’s specifications (ACTH: “ACTH ELISA”, RE 53081, IBL International GmbH Hamburg, Germany; cortisol: “Cortisol Saliva ELISA”, RE-52611, IBL International GmbH, Hamburg, Germany). Optical density was measured using a microtiter plate reader (Synergy H1 Multi-Mode Microplate Reader; BioTek Instruments Inc., Bad Friedrichshall, Germany). Detection limits were 1 pg/mL for ACTH and 0.003 μg/dL for CORT. Mean inter-assay and intra-assay CVs were 4.0% and 7.6% for ACTH and below 9.3% and 7.3% for CORT according to the manufacturer’s information. Due to technical problems, ACTH data were missing for 3 TSST participants and 5 PlacTSST participants, as well as CORT data for 2 TSST participants and 4 PlacTSST participants.

Renin–angiotensin–aldosterone system (RAAS): To assess endocrine parameters of the RAAS, we determined active renin (REN) and aldosterone (ALD) from EDTA plasma at 5 sampling timepoints (−1, +1, +10, +20, +30 min). We used commercial ELISAs according to the manufacturer’s specifications (renin: “Renin (active) ELISA”, RE 53321, IBL International GmbH, Hamburg, Germany; aldosterone: “Aldosterone ELISA”, RE-52301, IBL International GmbH, Hamburg, Germany). Optical density was measured using a microtiter plate reader (Synergy H1 Multi-Mode Microplate Reader, BioTek Instruments Inc., Bad Friedrichshall, Germany). Detection limits were 4.31 pg/mL for REN and <12.07 pg/mL for ALD. Mean inter- and intra-assay CVs were 5.7% and 5.2% for REN and 6.7% and 4.9% for ALD. We report missing data for REN for 3TSST participants and 2 PlacTSST participants as well as, for ALD, for 2 TSST participants and 1 PlacTSST participant.

### 2.6. Statistical Analyses

We used the statistic software SPSS (Version 28.0, IBM SPSS Statistics, Chicago, IL, USA). Data are presented as mean ± standard error of the mean (SEM). Tests were two-tailed with the significance level set at *p* < 0.05. Effect size parameters (*f*) were calculated from partial eta squared (η_p_^2^) using G*Power (Version 3.1.9.6, Heinrich Heine Universität Düsseldorf, Germany) and are reported where appropriate (effect size conventions η_p_^2^: 0.01 = small, 0.06 = medium, 0.14 = large; *f*: 0.10 = small, 0.25 = medium, 0.40 = large) [75]. We performed a priori power analyses using the statistical software G*Power for Macintosh (Version 3.1.9.6; Heinrich Heine University Düsseldorf, Germany). The required sample size to detect differences in immune cell count reactivity between TSST participants and PlacTSST participants, i.e., time-by-condition interactions in 5 (time) × 2 (condition) repeated measures analyses of variance (ANOVAs), exhibited conservatively expected small effect sizes of f = 0.10, with a power of (1 − β) = 0.80 or greater, α = 0.05, non-sphericity correction ε = 0.75, and an expected observed average correlation of repeated measures of r > 0.78 is N = 64. Prior to statistical analyses, all data were tested for normal distribution and homogeneity of variance using Kolmogorov–Smirnov and Levene’s tests. In case of violation of the normal distribution assumption, data were transformed using the natural logarithm. To account for violations of sphericity, we applied Huynh–Feldt corrections where appropriate.

Participants’ characteristics: To characterize our participants, we compared TSST participants and PlacTSST participants in terms of demographic and psychological variables, as well as in terms of baseline levels of immune cell counts and endocrine measures by calculating univariate analyses of variance (ANOVAs).

Reactivity to acute psychosocial stress induction as compared to placebo-psychosocial stress induction: To test whether the TSST, as compared to the PlacTSST, induced significant increases in immune cell counts, endocrine parameters, and BV changes, we calculated repeated measures ANOVAs with condition (TSST vs. PlacTSST) as independent variable and repeated immune cell count, endocrine parameter, or BV change measures as dependent variables, testing for time-by-condition interaction effects. Immune cell count analyses were corrected for potential stress hemoconcentration effects [13]. Notably, immune cell count analyses without control for stress hemoconcentration effects are reported in Appendix A. Moreover, to account for potential confounding effects of age and body mass index (BMI) on immune cell count and/or endocrine stress reactivity [20,76,77,78], we performed all AN(C)OVAs without and with controlling for age and BMI as covariates. Notably, BMI was calculated as BMI = body weight (kg)/body height squared (m^2^). We additionally repeated analyses controlling for chronic stress in addition to age and BMI, given the proposed effects of chronic stress on resting immune cell counts as well as on immune cell redistribution to acute stress [4]. Post-hoc testing comprised univariate ANOVAs with condition (TSST vs. PlacTSST) as independent variable and single measurement timepoints of immune cell counts, endocrine parameters, or BV change measures as dependent variable.

Reactivity for each condition separately: Moreover, we performed separate analyses for each condition (TSST, PlacTSST), to test for TSST-/PlacTSST-induced increases by means of repeated measures ANOVAs with repeated immune cell count, endocrine, or BV change measures as repeated dependent variables. Here, post-hoc testing comprised repeated measures ANOVAs with baseline and separate post-stress measurements as repeated dependent variables.

Mediation analyses: To statistically investigate the mechanisms underlying the psychosocial stress-induced immune cell count changes, we first tested for mediation of immediate immune cell count increases in reaction to psychosocial stress from baseline to peak (i.e., +1 min after TSST/PlacTSST cessation).Second, we tested for mediation of subsequent immune cell count decreases from +1 min to +10 min after TSST/PlacTSST cessation. We calculated TSST-/PlacTSST-induced increases in immune cell counts and endocrine parameters as the difference between immune cell count increase peak (i.e., +1 min after TSST/PlacTSST cessation) and baseline of the respective parameter. Subsequent decreases in immune cell counts and parallel changes in potential mediators were calculated as the difference between immune cell counts or mediator levels +10 min after TSST/PlacTSST cessation and immune cell count increase peak at +1 min.

To determine significant interaction effects (time-by-condition) in our repeated measures analyses with uncorrected immune cell counts, we performed mediation analyses using the PROCESS macro for SPSS [79]. PROCESS uses ordinary least squares regressions for parameter estimation and mediation effects are assessed based on the indirect effect of X (independent variable) on Y (dependent variable) through M(s) (mediator(s). The indirect effect is considered significant when the upper and lower bounds of the respective confidence interval (CI) do not contain zero. We employed bootstrapping with 10,000 samples together with heteroscedasticity consistent standard errors (HC3) [80] to compute 95% CI and inference statistics and report partially standardized indirect effects.

In all mediation models, we considered the condition as the dichotomous independent variable (TSST (1) vs. PlacTSST (0)) and immune cell count increases and subsequent decreases as the respective dependent variables. To prevent overfitting and to account for correlations between mediators, we applied the following procedure:First, we exploratorily tested for a mediating effect of TSST-/PlacTSST-induced immediate increases from baseline to +1 min after TSST/PlacTSST cessation of each potential mediator under study (i.e., changes in BV, EP, NEP, ACTH, CORT, REN, and ALD). Then, for main mediation analyses, we entered all identified mediators simultaneously, without and with additionally controlling for age and BMI as covariates. Second, we repeated the analyses described above, considering subsequent decreases in immune cell counts from +1 min to +10 min after TSST/PlacTSST cessation and simultaneous changes in potential mediators instead of increases.

## 3. Results

### 3.1. Participants’ Characteristics

The characteristics of our participants are presented in Table 1. The TSST participants and PlacTSST participants did not differ in any demographic, physiological, or psychological variables or in their baseline levels of immune and endocrine measures.

### 3.2. Reactivity to Acute Psychosocial Stress Induction as Compared to Placebo-Psychosocial Stress Induction

#### 3.2.1. Reactivity of Peripheral Blood Leukocyte Subpopulation Counts

**Figure 1:**
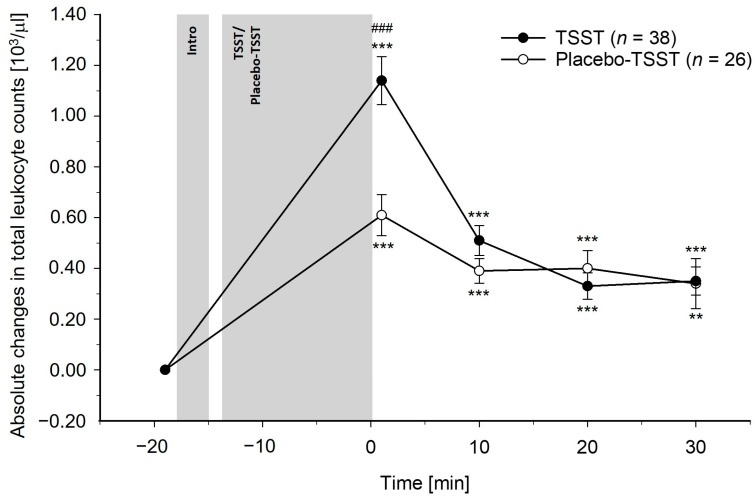
Absolute changes in total leukocyte counts in reaction to the TSST and the placebo-TSST, corrected for stress hemoconcentration effects. TSST = Trier social stress test. Asterisks indicate significant differences between baseline and later measurement timepoints within the two conditions, respectively, *** *p* < 0.001, ** *p* < 0.01. Hashtags indicate significant differences between TSST participants and placebo-TSST participants, ### *p* < 0.001.

**Figure 2:**
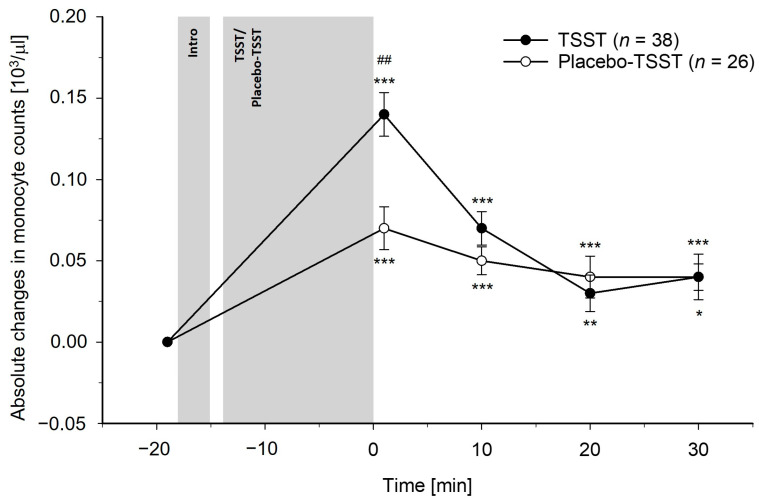
Absolute changes in monocyte counts in reaction to the TSST and the placebo-TSST, corrected for stress hemoconcentration effects. TSST = Trier social stress test. Asterisks indicate significant differences between baseline and later measurement timepoints within the two conditions, respectively, *** *p* < 0.001, ** *p* < 0.01, * *p* < 0.05. Hashtags indicate significant differences between TSST participants and placebo-TSST participants, ## *p* < 0.01.

**Figure 3:**
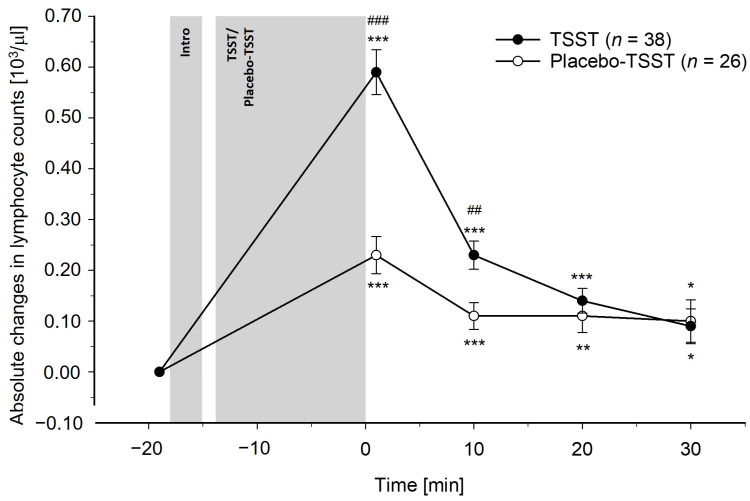
Absolute changes in lymphocyte counts in reaction to the TSST and the placebo-TSST, corrected for stress hemoconcentration effects. TSST = Trier social stress test. Asterisks indicate (borderline) significant differences between baseline and later measurement timepoints within the two conditions, respectively, *** *p* < 0.001, ** *p* < 0.01, * *p* < 0.05. Hashtags indicate significant differences between TSST participants and placebo-TSST participants, ### *p* < 0.001; ## *p* < 0.01.

**Figure 4:**
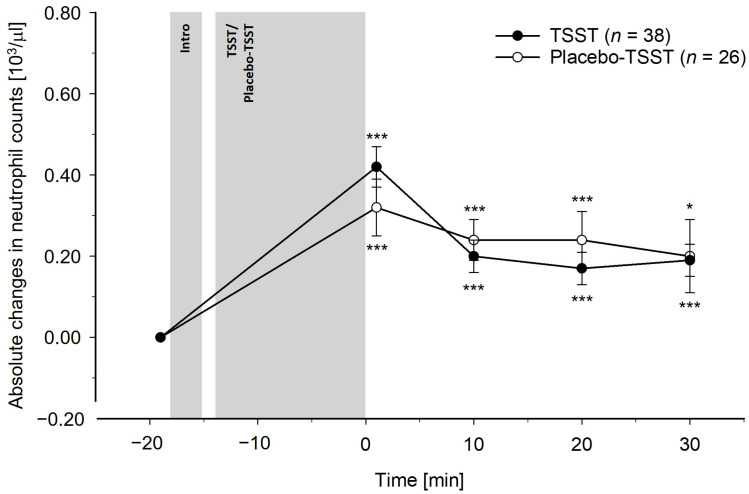
Absolute changes in neutrophil counts in reaction to the TSST and the placebo-TSST, corrected for stress hemoconcentration effects. TSST = Trier social stress test. Asterisks indicate (borderline) significant differences between baseline and later measurement timepoints within the two conditions, respectively, *** *p* < 0.001, * *p* < 0.05. There were no significant differences between TSST participants and placebo-TSST participants (*p*’s ≥ 0.12).

**Figure 5:**
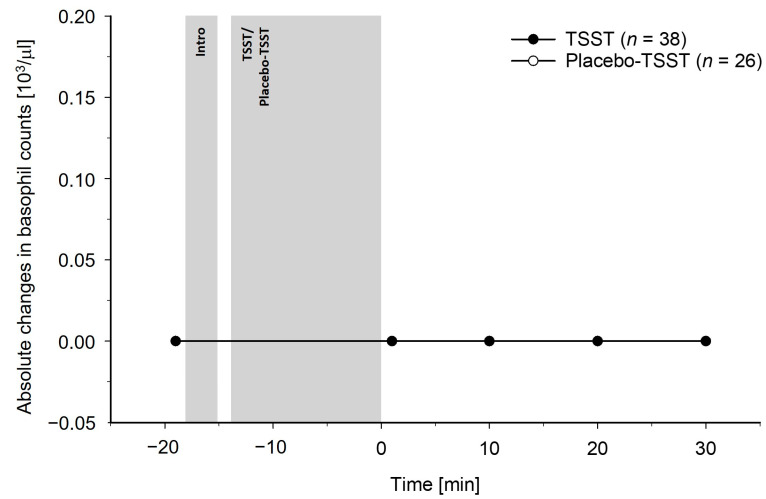
Absolute changes in basophil granulocyte counts in reaction to the TSST and the placebo-TSST, corrected for stress hemoconcentration effects. TSST = Trier social stress test. There were no significant differences between TSST participants and placebo-TSST participants (*p*’s ≥ 0.77).

**Figure 6:**
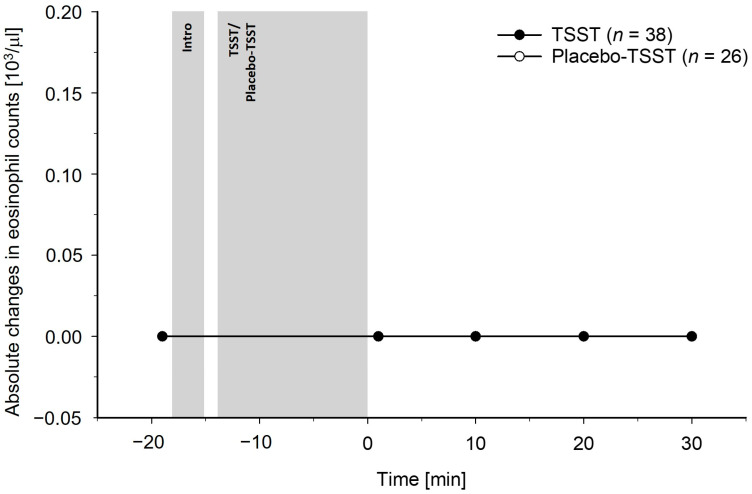
Absolute changes in eosinophil granulocyte counts in reaction to the TSST and the placebo-TSST corrected for stress hemoconcentration effects. TSST = Trier social stress test. There were no significant differences between TSST participants and placebo-TSST participants (*p*’s ≥ 0.86).

Differences between TSST and PlacTSST participants: The TSST induced greater increases in total leukocyte, monocyte, and lymphocyte counts as compared to the PlacTSST (interaction effects of time-by-condition: total leukocytes: *F*(3.28, 203.29) = 10.83, *p* < 0.001, η_p_^2^ = 0.15, *f* = 0.42; monocytes: *F*(3.59, 222.78) = 5.37, *p* < 0.001, η_p_^2^ = 0.08, *f* = 0.29; lymphocytes: *F*(2.88, 178.66) = 16.54, *p* < 0.001, η_p_^2^ = 0.21, *f* = 0.52). Controlling for potential confounders (i.e., age, BMI, and/or chronic stress) did not change these results (interaction effects of time-by-condition: *p*’s ≤ 0.001). Except for monocytes, where we observed a significant interaction effect of time-by-age (age and BMI: *F*(3.59, 215.74) = 2.63, *p* = 0.041, η_p_^2^ = 0.04, *f* = 0.20; age, BMI, and chronic stress: *p* = 0.11), there were neither significant main effects of age, BMI, chronic stress (*p*’s ≥ 0.07), nor other significant interaction effects of time-by-age, time-by-BMI, or time-by-chronic stress (*p*’s ≥ 0.19). There were no differences between conditions in the granulocyte count reactivity, without or with control for confounders (neutrophil granulocytes: *p*’s ≥ 0.12; eosinophil granulocytes: *p*’s ≥ 0.86; basophil granulocytes: *p*’s ≥ 0.77). Regarding age, BMI, and chronic stress, we did not observe significant main effects (*p*’s ≥ 0.37) or interaction effects of time-by-chronic stress (*p*’s ≥ 0.38). For eosinophil granulocytes, there were significant interaction effects of time-by-age (age and BMI: *F*(4, 240) = 2.76, *p* = 0.028, η_p_^2^ = 0.04, *f* = 0.20; age, BMI, and chronic stress: *F*(4, 220) = 2.17, *p* = 0.074, η_p_^2^ = 0.04, *f* = 0.20) and time-by-BMI (age and BMI: *F*(4, 240) = 2.72, *p* = 0.030, η_p_^2^ = 0.04, *f* = 0.20; age, BMI, and chronic stress: *F*(34, 220) = 2.80, *p* = 0.027, η_p_^2^ = 0.05, *f* = 0.23). These effects were not observed for the neutrophil granulocytes or basophil granulocytes (*p*’s ≥ 0.23). The reactivity of the peripheral blood leukocyte subpopulations to the TSST and the PlacTSST is depicted in Figure 1, Figure 2, Figure 3, Figure 4, Figure 5 and Figure 6.

Post-hoc testing regarding differences between conditions at separate timepoints revealed significant differences between the TSST participants and PlacTSST participants that occurred immediately, i.e., +1 min after TSST/PlacTSST cessation while controlling for baseline counts, but not at later timepoints for total leukocyte and monocyte counts (main effects of condition: total leukocytes: +1 min: *p* < 0.001; +10–30 min: *p*’s ≥ 0.22; monocytes: +1 min: *p* = 0.002; +10–30 min: *p*’s ≥ 0.25). For the lymphocyte counts, we observed significant differences between conditions +1 min and +10 min after TSST/PlacTSST cessation (main effects of condition: +1 min, +10 min: *p*’s ≤ 0.003; +20 min, +30 min: *p*’s ≥ 0.23).

Separate analyses in TSST participants and PlacTSST participants: Further testing comprised separate analyses in each condition, where we tested for significant changes in immune cell counts over time. We observed significant increases in reaction to both, TSST and PlacTSST, for total leukocyte, monocyte, and lymphocyte counts, as well as for neutrophil granulocyte counts (main effects of time: *p*’s < 0.001). The immune cell counts were (borderline) significantly elevated as compared to baseline at all post-stress measurement timepoints in both conditions for total leukocyte, monocyte, lymphocyte, and neutrophil granulocyte counts (main effects of time: total leukocytes: *p*’s ≤ 0.001; monocytes: *p*’s ≤ 0.011; lymphocytes: *p*’s ≤ 0.052; neutrophil granulocytes: *p*’s ≤ 0.013). There were no increases in basophil and eosinophil granulocytes neither in reaction to the TSST nor in reaction to the PlacTSST (main effects of time: *p*’s ≥ 0.43).

#### 3.2.2. Reactivity of Endocrine Parameters and Blood Volume Changes

Differences between TSST participants and PlacTSST participants: As expected and in line with our previous findings [54,66], the TSST induced greater increases as compared to the PlacTSST in all considered endocrine parameters, i.e., EP, NEP, ACTH, CORT, REN, and ALD (interaction effects time-by-condition: *p*’s ≤ 0.048, with age, BMI: *p*’s ≤ 0.067; with age, BMI, TICS: *p*’s ≤ 0.098; see Appendix A). Moreover, we observed greater BV decreases in reaction to the TSST as compared to the PlacTSST, as expected and in line with the literature [12,13] (*F*(4, 248) = 8.94, *p* < 0.001, η_p_^2^ = 0.13, *f* = 0.39; with age, BMI: *F*(4, 240) = 8.73, *p* < 0.001, η_p_^2^ = 0.13, *f* = 0.39; with age, BMI, TICS: *F*(4, 220) = 7.96, *p* < 0.001, η_p_^2^ = 0.12, *f* = 0.37). The TSST-/PlacTSST-induced changes in the BV and in endocrine parameters from baseline to +1 min as well as from +1 min to +10 min after TSST/PlacTSST cessation are depicted in Table 2.

Separate analyses in TSST participants and PlacTSST participants: Further analyses for both conditions separately revealed significant BV decreases in reaction to both the TSST and the PlacTSST (TSST: *F*(4, 148) = 100.27, *p* < 0.001, η_p_^2^ = 0.73, *f* = 1.64; PlacTSST: *F*(4, 100) = 23.27, *p* < 0.001, η_p_^2^ = 0.48, *f* = 0.96) as well as significant increases in all tested endocrine parameters (*p*’s ≤ 0.006), except for CORT. For CORT, we observed significant increases in reaction to the TSST (*p* < 0.001) as well as significant decreases in reaction to the PlacTSST (*p* < 0.001). For detailed results of the stress reactivity analyses of the endocrine parameters, please see Appendix A.

### 3.3. Mediation Analyses

#### 3.3.1. Mediation of Immune Cell Count Increases

First, we performed exploratory mediation analyses with the condition (TSST vs. PlacTSST) as the predictor, increases in the identified psychosocial stress-reactive leukocyte subpopulations (i.e., total leukocytes, monocytes, lymphocytes) as the criterion, and changes in BV, EP, NEP, ACTH, CORT, REN, or ALD from baseline to peak (+1 min) entered separately as potential mediators. Significant indirect effects were found: (1) for BV change in total leukocytes (ab = −0.40; 95% CI [−0.74; −0.13]) and monocytes (ab = −0.47; 95% CI [−0.85; −0.16]), but not in the total lymphocytes (95% CI [−0.52; 0.02]); (2) for EP increase in total leukocytes (ab = −0.53; 95% CI [−0.78; −0.34]), monocytes (ab = −0.57; 95% CI [−0.80; −0.35]), and lymphocytes (ab = −0.47; 95% CI [−0.70; −0.22]), and (3) for NEP increase in monocytes (ab = −0.39; 95% CI [−0.75; −0.04]) and lymphocytes (ab = −0.39; 95% CI [−0.71; −0.15]) but not in total leukocytes (95% CI [−0.62; 0.07]). There were no significant indirect effects for ACTH (95% CIs [lower limits (LLs) ≤ −0.46; upper limits (ULs) ≥ 0.17]), CORT (95% CIs [LLs ≤ −0.51; ULs ≥ 0.08]), REN (95% Cis [LLs ≤ −0.19; ULs ≥ 0.06]), or ALD (95% Cis [LLs ≤ −0.03; ULs ≥ 0.11]).

Second, in the main mediation analyses, we entered, for each significantly psychosocial stress-reactive immune cell count, all identified potential mediators of the exploratory analyses simultaneously. These were BV and EP changes for total leukocyte count increases, BV, EP, and NEP changes for monocyte count increases, and EP and NEP changes for lymphocyte count increases. EP increase was the only mediator that remained significant for all immune cell counts (total leukocytes: ab = −0.47; 95% CI [−0.75; −0.24]; monocytes: ab = −0.47; 95% CI [−0.75; −0.20]; lymphocytes: EP: ab = −0.43; 95% CI [−0.66; −0.11]). Neither BV (total leukocytes: 95% CI [−0.45; 0.06]; monocytes: 95% CI [−0.51; 0.03]) changes nor NEP (monocytes: 95% CI [−0.47; 0.18]; lymphocytes: 95% CI [−0.56; 0.05]) changes could be identified as mediating parameters of immune cell count increases in our main mediation analyses. Additional controlling for age and BMI did not significantly change these results (total leukocytes: EP: ab = −0.47; 95% CI [−0.79; −0.24]; BV: 95% CI [−0.45; 0.07]; monocytes: EP: ab = −0.49; 95% CI [−0.80; −0.23]; BV: 95% CI [−0.42; 0.07]; NEP: 95% CI [−0.49; 0.22]; lymphocytes: EP: ab = −0.44; 95% CI [−0.70; −0.09]; NEP: 95% CI [−0.56; 0.04]).

#### 3.3.2. Mediation of Immune Cell Count Decreases from +1 min to +10 min After TSST/PlacTSST Cessation

First, we again performed exploratory mediation analyses with the condition (TSST vs. PlacTSST) as the predictor, the immediate immune cell count decreases from +1 min to +10 min after TSST/PlacTSST cessation of identified psychosocial stress-reactive immune cell counts (i.e., total leukocytes, monocytes, lymphocytes) as the criterion, and changes in BV, EP, NEP, ACTH, CORT, REN, or ALD from +1 min to +10 min after TSST/PlacTSST cessation, entered separately, as potential mediators. Significant indirect effects were found: (1) for BV change in total leukocytes (ab = 0.46; 95% CI [0.16; 0.77]), monocytes (ab = 0.40; 95% CI [0.13; 0.72]) and lymphocytes (ab = 0.21; 95% CI [0.01; 0.53]), (2) for EP change in total leukocytes (ab = 0.52; 95% CI [0.32; 0.76]), monocytes (ab = 0.45; 95% CI [0.30; 0.80]), and lymphocytes (ab = 0.40; 95% CI [0.16; 0.64]), (3) for NEP change in leukocytes (ab = 0.36; 95% CI [0.10; 0.66]) and lymphocytes (ab = 0.41; 95% CI [0.17; 0.75]) but not in monocytes (95% CI [−0.12; 0.53]), (4) for CORT change in total leukocytes (ab = 0.28; 95% CI [0.05; 0.65]) and monocytes (ab = 0.31; 95% CI [0.06; 0.71]) but not in lymphocytes (95% CI [−0.06; 0.45]), and (5) for REN change in total leukocytes (ab = 0.19; 95% CI [0.05; 0.38]) and lymphocytes (ab = 0.21; 95% CI [0.04; 0.43]) but not in monocytes (95% CI [−0.03; 0.29]). There were no significant indirect effects for ACTH change (95% CIs [LLs ≤ −0.17; ULs ≥ 0.12]) or ALD change (95% CIs [LLs ≤ −0.03; ULs ≥ 0.20]).

Second, in the main analyses, we entered, for each significantly psychosocial stress-reactive blood cell count, all identified potential mediators of the exploratory analyses simultaneously. These were BV, EP, NEP, CORT, and REN changes for total leukocyte count decreases, BV, EP, and CORT changes for monocyte count decreases, and BV, EP, NEP, and REN changes for lymphocyte count decreases. With respect to total leukocyte count decreases, changes in the BV (ab = 0.31; 95% CI [0.04; 0.71]; with control for age and BMI: ab = 0.36; 95% CI [0.12; 0.74]), EP (ab = 0.42; 95% CI [0.17; 0.66]; with control for age and BMI: ab = 0.38; 95% CI [0.11; 0.62]) and REN (ab = 0.17; 95% CI [0.03; 0.37]; with control for age and BMI: ab = 0.17; 95% CI [0.04; 0.35]) remained as significant mediators, but none of the other exploratorily identified parameters, independent of age and BMI, did (NEP: 95% CI [−0.10; 0.49]; CORT: 95% CI [−0.38; 0.12]; with control for age and BMI: NEP: 95% CI [−0.18; 0.50]; CORT: 95% CI [−0.28; 0.19]). With respect to monocyte counts, the EP change was the only mediator that remained significant independent of age and BMI (EP: ab = 0.36; 95% CI [0.10; 0.77]; with age and BMI: EP: ab = 0.31; 95% CI [0.05; 0.73]). Neither BV nor CORT remained as a mediating parameter of immediate monocyte decreases in our main analysis (BV: 95% CI [−0.11; 0.56]; CORT: 95% CI [−0.21; 0.49]; with age and BMI: BV: 95% CI [−0.01; 0.61]; CORT: 95% CI [−0.011; 0.43]). With respect to lymphocyte count decreases, NEP (ab = 0.24; 95% CI [0.01; 0.53]) and REN (ab = 0.18; 95% CI [0.01; 0.41]) changes emerged as the only mediators without controlling for age and BMI, while BV and EP did not (BV: 95% CI [−0.05; 0.58]; EP: 95% CI [−0.03; 0.57]). Controlling for age and BMI, BV (ab = 0.26; 95% CI [0.04; 0.61]) and REN (ab = 0.18; 95% CI [0.02; 0.38]) changes emerged as remaining mediators but not EP (95% CI [−0.06; 0.53]) or NEP (95% CI [−0.01; 0.52]) changes.

## 4. Discussion

Here, we investigated for the first time the specifically acute psychosocial stress-induced redistribution of peripheral blood leukocyte subpopulations by considering an active placebo-psychosocial stress control condition. The latter is highly comparable to the psychosocial stress condition but without the psychosocial stress-inducing elements of social evaluation and uncontrollability. Therefore, it controls for secondary effects of the physical or cognitive demands of the stress task and allows researchers to explicitly attribute observed differences between the reactivity to the TSST and that to the PlacTSST to psychosocial stress. We assessed total leukocyte, lymphocyte, and monocyte counts as well as neutrophil, eosinophil, and basophil granulocyte counts before and repeatedly after psychosocial stress/placebo-psychosocial stress and corrected for stress hemoconcentration, given the potentially confounding of BV changes on immune cell count measurements [12,13]. Moreover, inspired by previous research that points to endocrine mechanisms underlying the immune cell count redistribution which results from acute stress (e.g., [5,9,19,31,33,46,56,57,58,59,60,61,62,63,64], we investigated the mechanisms underlying the observed increases in total leukocyte, monocyte, and lymphocyte counts in reaction to acute psychosocial stress specifically. In this regard, we tested whether TSST-induced changes in endocrine parameters (EP, NEP, ACTH, CORT, REN, ALD) and BV would mediate the observed parallel immediate total leukocyte, monocyte, and lymphocyte count increases and the subsequent decreases within the recovery period after the peak from +1 min to +10 min after TSST cessation.

The first main finding of our study is that acute psychosocial stress, as compared to the active placebo-psychosocial stress control condition, induced significantly greater increases in total leukocyte, monocyte, and lymphocyte counts. These effects were of medium to large statistical effect sizes and were observed both with and without correction for stress-induced BV changes, as well as with and without controlling for age, BMI, and chronic stress as potential confounders. Contrary to our expectations, there were no differences in the reactivity of neutrophil granulocytes between the psychosocial stress condition and the placebo-psychosocial stress condition. As the placebo-psychosocial stress control condition is highly comparable to the psychosocial stress condition with respect to setting and task, except for the psychosocial stress-inducing elements of social-evaluation and uncontrollability, the observed higher total leukocyte, monocyte, and lymphocyte count increases observed in our TSST participants can specifically be attributed to the psychosocial stress-inducing components of the psychosocial stress condition and are not secondary to the physical or cognitive demands of the task [51,52]. Notably, the observed increases in total leukocyte, monocyte, and lymphocyte counts following acute psychosocial stress are in line with previous studies that assessed immune cell count reactivity to moderate or strong mental stressors, as compared to non-stress baseline measurements, while controlling for stress hemoconcentration effects [29,31,37,39,42]. Interestingly, we could not observe differences in the neutrophil granulocyte redistribution between the psychosocial stress condition and the active placebo-psychosocial stress condition when controlling for stress hemoconcentration effects. However, the neutrophil granulocyte counts increased significantly in reaction to both the TSST and the PlacTSST. This suggests a rapid non-psychosocial stress-specific increase in circulating neutrophil granulocytes that already occurs in reaction to situations with a minor potential of threat or harm and a consequently lower probability of warranting an immune response in situations such as our active placebo-psychosocial stress control condition. In line with this reasoning, significant increases in neutrophil granulocyte counts have been frequently observed in reaction to mental stressors of all intensities, including mild mental stress, in studies with [31,39] and even in studies without controlling for stress hemoconcentration effects [23,30,43,45,47]. Potential explanations for these observed psychosocial stress-specific findings may relate to functional differences of the different immune cell populations. Notably, both, neutrophil granulocytes and monocytes are phagocytic immune cells that play a crucial role, especially in the early phase, in the innate inflammatory response [81,82]. However, in contrast to neutrophil granulocytes, monocytes can additionally act as competent antigen-presenting cells of particular importance for activation of the adaptive immune response carried out by lymphocytes [81,83,84]. Therefore, our results may suggest that psychosocial stress, i.e., strong mental stress, specifically prepares one for activation of both the adaptive and innate immune systems while minor non-psychosocial stress-induced activation, e.g., resulting from non-psychosocial stress-inducing activities such as standing and speaking out loud, can only prepare for the activation of the innate immune system, presumably to avoid immune system overactivation. Notably, previous infusion and correlational studies point to catecholamines as a mediator underlying the stress-induced immune cell count changes [19,31,46,56,57,58,59,60,61,62,63,64]. Extending these findings, EP was identified as the main mediator of the higher lymphocyte and monocyte count increases observed in reaction to the TSST as compared to the PlacTSST in our mediation analyses. Moreover, both innate and adaptive immune cells express adrenergic receptors, enabling them to respond to catecholamines [55,85,86]. We therefore speculate that differences in the density and sensitivity of adrenergic receptors between immune cell subpopulations [85,87,88] may relate to the differences in the reactivity to the TSST as compared to the PlacTSST between leukocyte subpopulations, i.e., higher increases in monocytes and lymphocytes in reaction to the TSST as compared to the PlacTSST, as opposed to comparable increases in neutrophil granulocytes in reaction to the TSST and to the PlacTSST.

There were no differences in the basophil and eosinophil granulocyte count reactivity between the psychosocial stress and the placebo-psychosocial stress control conditions. Moreover, neither psychosocial stress nor placebo-psychosocial stress was capable of inducing increases in basophil and eosinophil granulocyte counts. This is in line with the only previous study investigating basophil and eosinophil granulocyte count changes following strong mental stress while controlling for stress hemoconcentration effects, notably without any control condition [31]. This non-reactivity to psychosocial as well as mild mental and/or physical stress may relate to the minor importance of basophil and eosinophil granulocytes in the case of stress-related harm or injury, as their main functions are non-acute defense functions such as allergy and parasitic infections [89,90].

Building upon the work of Dhabhar, McEwen, and colleagues (e.g., [5,9,91,92]) regarding the role of stress hormones in mediating, i.e., inducing, the stress-induced redistribution of immune cells), we investigated endocrine- and BV-related mechanisms underlying the observed greater redistribution of total leukocyte, monocyte, and lymphocyte counts in reaction to the TSST as compared to the PlacTSST. Our second main finding is that our mediation analysis results suggest that these immediate psychosocial stress-induced increases +1 min after TSST cessation are mediated, i.e., induced, by parallel increases in EP. Notably, in our exploratory mediation analyses, where we entered the mediators separately, NEP also emerged as a significant mediator for stress-induced monocyte and lymphocyte increases as well as BV changes for total leukocyte and monocyte counts, but lost significance when we considered further mediators, in particular EP. Given our results, CORT, ACTH, REN, and ALD do not seem to play a role in mediating psychosocial stress-induced increases in immune cell counts. Subsequent decreases in immune cell counts from +1 to +10 min after TSST cessation were also mediated by parallel EP decreases with respect to total leukocyte and monocyte decreases, and by decreases in REN and BV changes as (additional) mediators for total leukocyte and lymphocyte decreases when controlling for age and BMI. Similar to immune cell count increases, NEP emerged as a significant mediator in our exploratory mediation analyses where we entered mediators separately, but lost significance when we considered further mediators. There were no effects of ALD and ACTH. The observed mediating role of EP in immediate psychosocial stress-induced immune cell count increases is in line with infusion studies which report increases in total leukocyte, monocyte, or lymphocyte counts following EP infusion [57,58,60,61,62,63,64]. Also, one correlational study found positive associations between EP changes and increases in total leukocytes, as well as increases in lymphocyte subpopulations in reaction to a series of different mental stressors of mild or moderate intensity [46]. However, other studies failed to find associations between increases in total leukocyte, monocyte, and lymphocyte counts and parallel EP increases [19,31], but reported associations between stress-induced immune cell count increases and NEP or CORT [19,31,44]. This divergence in findings may relate to the methods applied for statistical analysis. In our study, we conducted mediation analyses, testing for mediators of stress-induced immune cell count increases, including the simultaneous testing of multiple potential mediating variables. Previous studies, in contrast, tested for linear associations between immune cell count increases following stress and changes in single endocrine parameters. In line with previous findings [19,29,31], in our exploratory mediation analyses where we entered mediators separately, NEP similarly emerged as a significant mediator of stress-induced monocyte and lymphocyte increases as well as BV changes for total leukocytes and monocytes, but lost significance when we considered further mediators, in particular EP.

This is the first study that investigated the mediating role of endocrine parameters beyond CORT and NEP, namely REN, ALD, ACTH, and EP, as well as that of BV during the immediate recovery period after psychosocial stress-induced immune cell count increases, i.e., from +1 min to +10 min after TSST cessation. Our results indicate that immune cell count decreases from +1 to +10 min after TSST cessation are mediated by parallel changes in EP with respect to total leukocyte and monocyte counts, and by changes in REN and BV as (additional) mediators for total leukocyte and lymphocyte decreases when controlling for age and BMI. Notably, there is one study that examined associations between monocyte, lymphocyte, and granulocyte counts changes from +10 to +60 min after TSST cessation and increases (in terms of changes from baseline to the individual peak) or decreases (in terms of changes from individual peak to individual minimum value in the time period from +30 to +120 min after TSST cessation) in CORT and NEP [33]. In this study, there were significant correlations between changes in lymphocyte and monocyte counts from +10 to +60 min after TSST cessation and cortisol increases as well as decreases. As opposed to this study [33], in our study, changes in lymphocyte or granulocyte numbers from +1 min to +10 min after TSST/PlacTSST cessation were not mediated by parallel changes in CORT in our main analyses with parallel testing of several mediators. Notably, in our exploratory mediation analyses where mediators were tested separately, CORT emerged as a mediator for changes in the total leukocyte and monocyte counts from +1 min to +10 min after TSST/PlacTSST cessation. In this regard, it should be noted that, given the (about 10 min) retarded stress-induced increases in salivary CORT as compared to those in plasma or serum CORT, in addition to the prolonged cortisol stress recovery interval of about 60 min [16], the investigation period of our mediation analysis of up to +10 min after TSST/PlacTSST cessation may have been too short to detect CORT effects on stress-induced immune cell count redistribution. This may have compromised our results regarding the effects of CORT on stress-induced immune cell count changes. Nevertheless, we interpret the observed decreases in immune cell counts after TSST cessation to likely reflect a traffic back to storage organs, as the TSST, unlike physically dangerous stress situations with injury potential, presumably requires no immune response. We speculate that the effects of REN on immune cell distribution and functioning relate to the role of REN in the regulation of extracellular volume [93]. More precisely, the recovery of stress-induced plasma REN increases occurs within 10–20 min after stress cessation [66]. We speculate that the concomitant normalization of the extracellular volume may serve as an “all-clear signal”, leading to the trafficking out of the blood back to storage organs, as no action was required, thereby mediating the decreases in total leukocyte and lymphocyte counts. The mediating role of BV changes in immune cell count decreases after stress is in line with the proposed effects of stress hemoconcentration on immune cell count stress reactivity [12,13,29].

Our findings may have clinical implications. The immediate redistribution of immune cell counts in reaction to acute psychosocial stress allows for rapid immune responses to impending challenges in case of injury, tissue damage, or antigen or pathogen entry [9,11]. In light of the mediating roles of EP, REN, and BV in the immediate immune cell count’s reactivity to psychosocial stress, alterations in the neuroendocrine stress reactivity, as, for instance, observed in chronically stressed individuals or individuals with psychiatric or somatic diseases or disease risk, may affect the immediate immune cell count reactivity to and recovery from psychosocial stress. Consequences may relate to a poor or slower recovery of stress-related injuries or infections and may further add to adverse health consequences [79,94].

The strengths of our study include, first, the combined use of the TSST and the PlacTSST, which allows to control for secondary physical and cognitive effects of the task (i.e., speaking and calculating) and setting (e.g., standing alone in an empty room, orthostatic effects) and, thus, to specifically attribute reactivity differences to psychosocial stress [53,54]. It should be noted that, in contrast to a passive resting and, thus, non-stress control condition, our active placebo-psychosocial stress control condition likely induced minor physical and/or mental stress. Second, by controlling for stress hemoconcentration effects in our analyses, we followed the state-of-the-art recommendations for research on changes in whole blood BMs in reaction to events associated with plasma alterations such as e.g., mental stress [13]. Third, examining the mechanisms underlying the psychosocial stress-induced immune cell redistribution using mediation analysis allows for a more comprehensive investigation as compared to correlational or infusion studies. Our study also has limitations. First, the generalizability of our findings is restricted to healthy young men and strong mental stress. Second, our investigation of underlying endocrine and hemoconcentration mechanisms was based on mediation analyses and requires confirmation from studies that allow for causality testing. Third, the chosen sampling timepoints and period as well as the assessment methods of the endocrine parameters may have affected our results.

## 5. Conclusions

Taken together, we found evidence for a specifically psychosocial stress-induced redistribution of certain peripheral blood leukocyte subpopulations, i.e., monocytes and lymphocytes. Our findings, thereby, on the one hand, confirm the results of previous studies that assessed monocyte or lymphocyte changes to moderate or strong mental stressors as compared to non-stress baseline measurements, notably without a control condition, while controlling for stress hemoconcentration effects [29,31,37,39,42]. On the other hand, based on our placebo control condition, that controlled for potential unspecific and non-psychosocial stress effects, our findings extend these previous findings to identify effects that are specific to psychosocial stress. Interestingly, differing from previous findings, our findings suggest that neutrophil granulocytes seem to be highly reactive, as they respond with immediate increases in their circulating numbers to both psychosocial stress and active placebo-stress. This points to an unspecific response of neutrophil granulocytes, even to situations with a low probability of threat or harm, such as our placebo-psychosocial stress condition, and which is thus not specific for psychosocial stress. Moreover, our study sheds further light on the mechanisms underlying the psychosocial stress-induced redistribution of peripheral blood leukocyte subpopulations in terms of immediate increases from baseline to +1 min after stress cessation and subsequent immediate decreases from +1 min to +10 min after stress cessation. Our results point to a major role of EP in immediate immune cell count increases in reaction to psychosocial stress as well as in subsequent decreases from +1 to +10 min after stress cessation, and of plasma REN and BV changes in immune cell count decreases from +1 to +10 min after stress cessation. The generalizability of our findings to populations other than healthy young men, as well as their mechanistic causality and clinical implications, remains to be elucidated in future studies.

## Figures and Tables

**Table 1:** Participants’ characteristics, including baseline levels of physiological parameters.

	TSST Participants (*n* = 38)	PlacTSST Participants (*n* = 26)	*p*
Age [years]	23.32 ± 0.40 (18–28)	23.38 ± 0.48 (20–29)	0.91
BMI [kg/m^2^]	24.03 ± 0.52 (18.37–33.19)	23.43 ± 0.45 (20.48–28.55)	0.41
TICS-CSSS	17.97 ± 1.65, *n* = 35 (3–42)	17.16 ± 1.77, *n* = 25 (6–40)	0.74
Baseline counts and levels			
MAP [mmHg]	90.38 ± 1.30 (74.33–109.00)	93.36 ± 1.66 (79.83–107.67)	0.16
Total leukocyte counts (10^3^/μL)	5.08 ± 0.17 (3.10–7.71)	5.52 ± 0.24 (3.60–8.41)	0.12
Monocyte counts (10^3^/μL)	0.40 ± 0.02 (0.21–0.60)	0.42 ± 0.06 (0.23–0.79)	0.37
Neutrophil granulocyte counts (10^3^/μL)	3.38 ± 0.14 (1.80–5.01)	3.57 ± 0.21 (2.40–6.17)	0.12
Basophil granulocyte counts (10^3^/μL)	0.03 ± 0.00 (0.01–0.08)	0.03 ± 0.00 (0.01–0.06)	0.40
Eosinophil granulocyte counts (10^3^/μL)	0.11 ± 0.01 (0.02–0.33)	0.10 ± 0.02 (0.01–0.44)	0.60
Lymphocyte counts (10^3^/μL)	1.35 ± 0.05 (0.85–2.32)	1.39 ± 0.08 (0.67–2.13)	0.67
Plasma epinephrine (pg/mL)	22.30 ± 2.49, *n* = 33 (8.00–67.00)	22.44 ± 1.90, *n* = 23 (8.30–39.90)	0.97
Plasma norepinephrine (pg/mL)	290.10 ± 16.18, *n* = 33 (148.30–605.90)	297.60 ± 25.57, *n* = 23 (166.00–624.80)	0.80
Plasma ACTH (pg/mL)	45.03 ± 7.71, *n* = 35 (4.87–270.97)	34.10 ± 6.08, *n* = 21 (3.67–111.11)	0.33
Salivary Cortisol (nmol/L)	5.45 ± 0.42, *n* = 36 (1.68–12.63)	6.44 ± 0.80, *n* = 22 (1.57–13.86)	0.23
Plasma Renin (pg/mL)	30.61 ± 2.48, *n* = 35 (8.16–81.04)	25.00 ± 3.31, *n* = 24 (5.98–81.96)	0.17
Plasma Aldosterone (pg/mL)	112.55 ± 12.31, *n* = 36 (29.28–284.27)	108.36 ± 13.44, *n* = 25 (39.87–285.44)	0.82

Note: values are means ± standard error of the mean (range). BMI = body mass index; *n* = sample size, deviating sample sizes of parameters are indicated; MAP = mean arterial blood pressure, calculated from two resting blood pressure measurements as 2/3 diastolic blood pressure + 1/3 systolic blood pressure; PlacTSST = placebo version of the Trier social stress test; TICS-CSSS = Chronic Stress Screening Scale of the Trier Inventory for Chronic Stress; TSST = Trier social stress test.

**Table 2:** TSST-/PlacTSST-induced changes from baseline to +1 min after TSST/PlacTSST cessation and from +1 min to +10 min after TSST/PlacTSST cessation in endocrine parameters and blood volume change.

		Baseline to +1 min After TSST/PlacTSST Cessation	*p*	+1 min to +10 min After TSST/PlacTSST Cessation	*p*
Blood volume (%)	TSST	−4.26 ± 0.31 (−9.33–1.25)	**<0.001**	7.70 ± 0.59 (−2.80–18.17)	**<0.001**
	PlacTSST	−2.44 ± 0.35 (−6.33–1.30)		4.69 ± 0.57 (−2.55–11.71)	
Plasma epinephrine (pg/mL)	TSST (*n* = 33)	56.35 ± 9.53 (0.90–283.70)	**<0.001**	−45.38 ± 8.60 (−264.80–−1.90)	**<0.001**
	PlacTSST (*n* = 23)	11.04 ± 3.05 (−8.10–43.80)		−6.57 ± 2.25 (−33.00–10.70)	
Plasma norepinephrine (pg/mL)	TSST (*n* = 33)	214.95 ± 19.28 (31.90–551.80)	**<0.001**	−184.55 ± 16.37 (−479.70–−49.10)	**<0.001**
	PlacTSST (*n* = 23)	74.48 ± 9.15 (−3.90–158.40)		−79.37 ± 10.57 (−176.80–38.00)	
Plasma ACTH (pg/mL)	TSST (*n* = 35)	23.35 ± 3.14 (−9.22–78.53)	**<0.001**	−9.89 ± 1.38 (−31.21–12.13)	**<0.001**
	PlacTSST (*n* = 21)	2.79 ± 1.33 (−10.42–15.22)		−1.98 ± 1.79 (−26.80–19.46)	
Salivary Cortisol (nmol/L)	TSST (*n* = 36)	6.74 ± 0.84 (−2.51–17.94)	**<0.001**	4.20 ± 1.01 (−3.52–29.42)	**<0.001**
	PlacTSST (*n* = 22)	0.50 ± 0.88 (−7.82–13.00)		−0.01 ± 0.41 (−5.30–3.23)	
Plasma renin (pg/mL)	TSST (*n* = 35)	11.10 ± 2.24 (−4.07–45.14)	**0.032**	−8.49 ± 2.07 (−45.72–14.89)	**0.018**
	PlacTSST (*n* = 24)	4.80 ± 1.08 (−9.45–15.30)		–2.09 ± 0.95 (−9.22–10.73)	
Plasma aldosterone (pg/mL)	TSST (*n* = 36)	48.06 ± 5.87 (5.91–156.81)	**0.095**	11.38 ± 3.52 (−30.17–75.12)	**0.19**
	PlacTSST (*n* = 25)	32.24 ± 7.34 (−33.55–132.76)		5.01 ± 2.68 (−24.25–28.03)	

Note: values are means ± standard error of the mean (range). ACTH = adrenocorticotropic hormone; TSST = Trier social stress test; PlacTSST = placebo version of the TSST; *n* = sample size, deviating sample sizes of parameters are indicated; statistically significant results are highlighted in bold.

**Figure A1:**
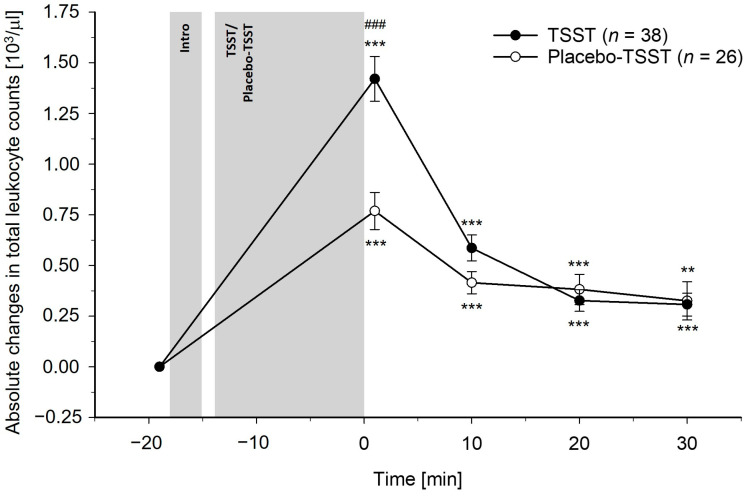
Absolute changes in total leukocyte counts in reaction to the TSST and the placebo-TSST without correction for stress hemoconcentration effects. TSST = Trier social stress test. Asterisks indicate significant differences between baseline and later measurement timepoints within the two conditions, respectively, *** *p* < 0.001, ** *p* < 0.01. Hashtags indicate significant differences between TSST participants and placebo-TSST participants, ### *p* < 0.001.

**Figure A2:**
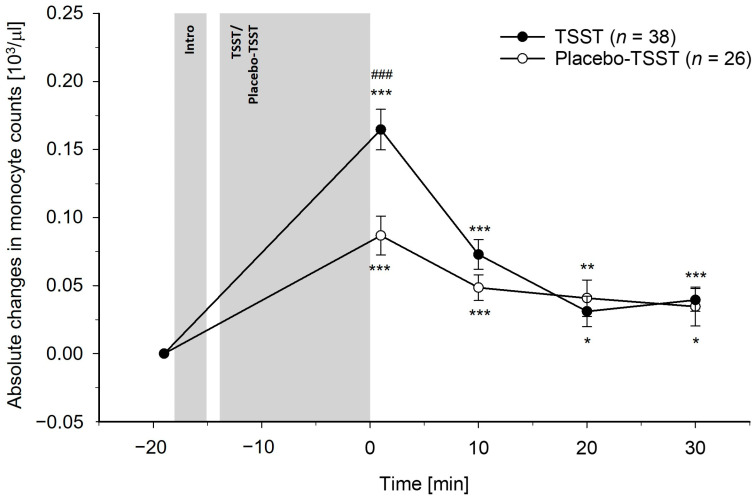
Absolute changes in monocyte counts in reaction to the TSST and the placebo-TSST without correction for stress hemoconcentration effects. TSST = Trier social stress test. Asterisks indicate significant differences between baseline and later measurement timepoints within the two conditions, respectively, *** *p* < 0.001, ** *p* < 0.01, * *p* < 0.05. Hashtags indicate significant differences between TSST participants and placebo-TSST participants, ### *p* < 0.001.

**Figure A3:**
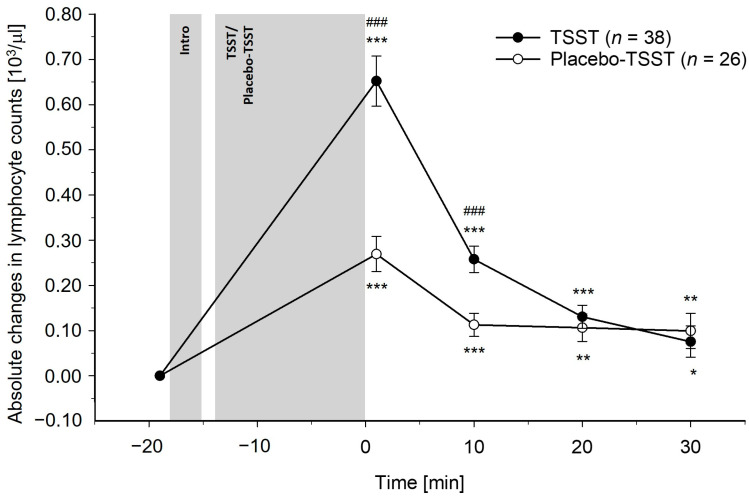
Absolute changes in lymphocyte counts in reaction to the TSST and the placebo-TSST without correction for stress hemoconcentration effects. TSST = Trier social stress test. Asterisks indicate (borderline) significant differences between baseline and later measurement timepoints within the two conditions, respectively, *** *p* < 0.001, ** *p* < 0.01, * *p* < 0.10. Hashtags indicate significant differences between TSST participants and placebo-TSST participants, ### *p* < 0.001.

**Figure A4:**
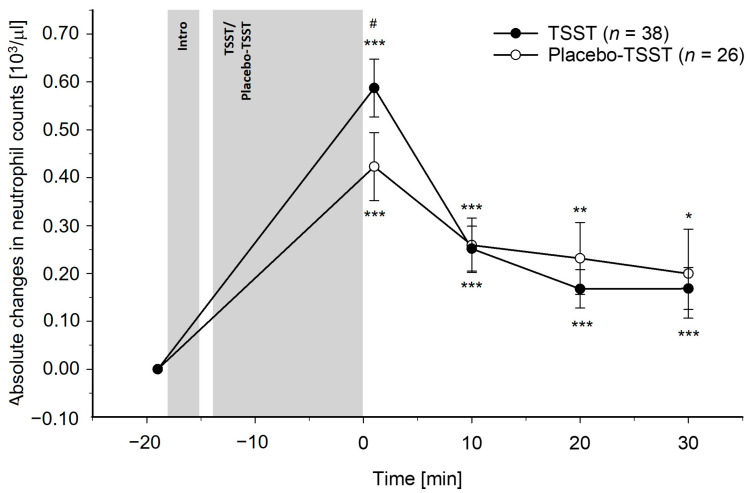
Absolute changes in neutrophil granulocyte counts in reaction to the TSST and the placebo-TSST without correction for stress hemoconcentration effects. TSST = Trier social stress test. Asterisks indicate significant differences between baseline and later measurement timepoints within the two conditions, respectively, *** *p* < 0.001, ** *p* < 0.01, * *p* < 0.05. Hashtags indicate significant differences between TSST participants and placebo-TSST participants, # *p* < 0.05.

**Figure A5:**
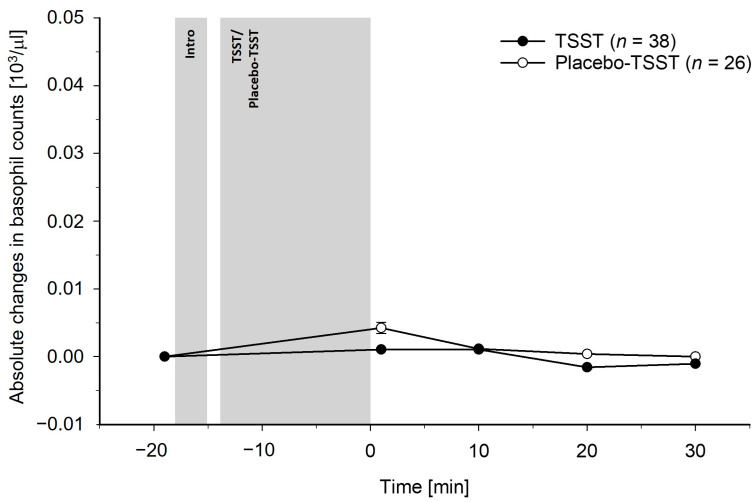
Absolute changes in basophil granulocyte counts in reaction to the TSST and the Placebo-TSST without correction for stress hemoconcentration effects. TSST = Trier social stress test. There were no significant differences between TSST participants and placebo-TSST participants.

**Figure A6:**
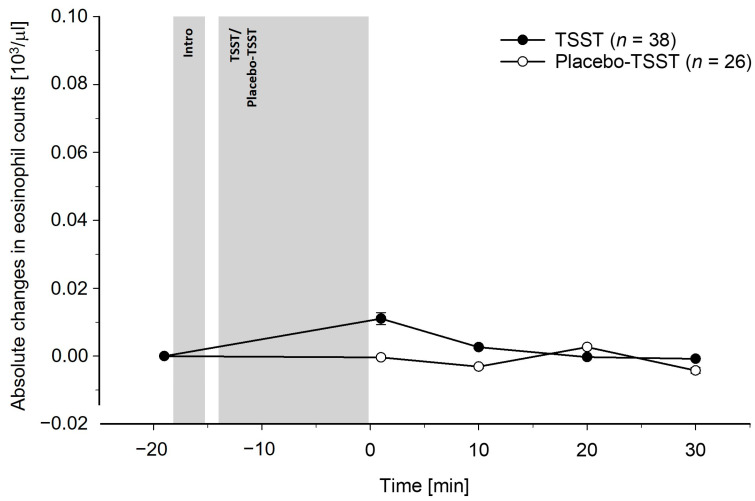
Absolute changes in eosinophil granulocyte counts in reaction to the TSST and the placebo-TSST without correction for stress hemoconcentration effects. TSST = Trier social stress test. There were no significant differences between TSST participants and placebo-TSST participants.

## Data Availability

Dataset available on request from the authors.

## Appendix A

### Appendix A.1. Supplementary Results

#### Appendix A.1.1. Reactivity of Immune Cell Counts Without Correction for Stress Hemoconcentration Effects to Acute Psychosocial Stress Induction as Compared to Placebo-Psychosocial Stress Induction

Differences between TSST participants and PlacTSST participants: The TSST induced greater increases in total leukocyte, monocyte, lymphocyte, and neutrophil granulocyte counts as compared to the PlacTSST (interaction effects of time-by-condition: total leukocytes: *F*(3.15, 195.36) = 14.58, *p* < 0.001, η_p_^2^ = 0.19, *f* = 0.48; monocytes: *F*(3.55, 219.97) = 6.59, *p* < 0.001, η_p_^2^ = 0.10, *f* = 0.33; neutrophil granulocytes: *F*(3.17, 196.25) = 3.69, *p* = 0.011, η_p_^2^ = 0.06, *f* = 0.25; lymphocytes: *F*(2.97, 183.96) = 19.72, *p* < 0.001, η_p_^2^ = 0.24, *f* = 0.56; see Figure A1, Figure A2, Figure A3 and Figure A4). There were no differences between the TSST participants and PlacTSST participants in eosinophil or basophil granulocyte counts (*p*’s ≥ 0.73; see Figure A5 and Figure A6).

Post-hoc testing for differences between the TSST participants and PlacTSST participants at separate timepoints revealed significant differences between conditions immediately, i.e., +1 min after TSST/PlacTSST cessation while controlling for baseline counts, but not at later timepoints for total leukocyte, monocyte, and neutrophil granulocyte counts (main effects of condition: total leukocytes: +1 min: *p* < 0.001; +10–30 min: *p*’s ≥ 0.099; monocytes: +1 min: *p* < 0.001; +10–30 min: *p*’s ≥ 0.19; neutrophil granulocytes: +1 min: *p* = 0.041; +10–30 min: *p*’s ≥ 0.26). With respect to lymphocyte count, we observed significant differences between the conditions both +1 min and +10 min after TSST/PlacTSST cessation (+1 min, +10 min: *p*’s < 0.001; +20 min, +30 min: *p*’s ≥ 0.24).

Separate analyses in TSST participants and PlacTSST participants: Further testing comprised separate analyses for each condition, where we tested for significant changes in the immune cell counts over time. We observed significant increases in reaction to both the TSST and the PlacTSST, for total leukocyte, monocyte, lymphocyte, and neutrophil granulocyte counts (main effects of time: *p*’s < 0.001). The immune cell counts were (marginal) significantly elevated as compared to baseline at all post-stress measurement timepoints in both conditions for total leukocyte, monocyte, lymphocyte, and neutrophil granulocyte counts (main effects of time: total leukocytes: *p*’s ≤ 0.001; monocytes: *p*’s ≤ 0.012; lymphocytes: *p*’s ≤ 0.092; neutrophil granulocytes: *p*’s ≤ 0.016). With respect to eosinophil granulocyte counts, we observed significant increases in reaction to the TSST (main effect of time: *F*(4, 148) = 3.10, *p* = 0.017, η_p_^2^ = 0.08, *f* = 0.29), with increased eosinophil granulocyte counts being observed +1 min after TSST cessation (main effect of time: *p* = 0.012) but not at later timepoints (main effects of time: *p*’s ≥ 0.39). In response to the PlacTSST, there were no changes in the eosinophil granulocyte counts (main effect of time: *p* = 0.58). There were no changes in the basophil granulocytes counts neither in reaction to the TSST nor to the PlacTSST (main effects of time: *p*’s ≥ 0.27).

#### Appendix A.1.2. Reactivity of Endocrine Parameters and Blood Volume Changes to Acute Psychosocial Stress Induction as Compared to Placebo-Psychosocial Stress Induction

Blood volume change: The TSST induced greater BV decreases in the TSST participants as compared to the PlacTSST in PlacTSST participants (interaction effect time-by-condition: *F*(4, 248) = 8.94, *p* < 0.001, η_p_^2^ = 0.13, *f* = 0.39). The effect was independent of potential confounders (interaction effects of time-by-condition: with age, BMI: *F*(4, 240) = 8.73, *p* < 0.001, η_p_^2^ = 0.13, *f* = 0.39; with age, BMI, TICS: *F*(4, 220) = 7.96, *p* < 0.001, η_p_^2^ = 0.12, *f* = 0.37). Post-hoc testing revealed significant differences between the two conditions +1 and +10 min after TSST/PlacTSST cessation (*p*’s ≤ 0.023) but not +20 or +30 min after TSST/PlacTSST cessation (*p*’s ≥ 0.26). Considering both conditions separately, we observed significant BV decreases in reaction to both the TSST (main effect of time: *F*(4, 148) = 100.27, *p* < 0.001, η_p_^2^ = 0.73, *f* = 1.64) and the PlacTSST (main effect of time: *F*(4, 100) = 23.27, *p* < 0.001, η_p_^2^ = 0.48, *f* = 0.96). While the TSST participants exhibited decreases in BV as compared to baseline +1 and +10 min after TSST cessation (*p*’s ≤ 0.001) and BV + 30 min after TSST cessation (*p* = 0.005), with no difference to baseline at +20 min (*p* = 0.41), the BV was decreased only immediately after PlacTSST cessation (+1 min: *p* < 0.001; +10–+30 min: *p*’s ≥ 0.18).

Epinephrine: The TSST induced higher plasma EP increases as compared to the PlacTSST (interaction effect time-by-condition: *F*(1.54, 83.11) = 27.41, *p* < 0.001, η_p_^2^ = 0.34, *f* = 0.72). Controlling for potential confounders did not significantly change these results (interaction effects of time-by-condition: with age, BMI: *F*(1.57, 81.47) = 26.05, *p* < 0.001, η_p_^2^ = 0.33, *f* = 0.70; with age, BMI, TICS: *F*(1.61, 80.47) = 25.14, *p* < 0.001, η_p_^2^ = 0.34, *f* = 0.72). Post-hoc testing revealed significant differences between TSST participants and PlacTSST participants +1 min and + 10 min after TSST/PlacTSST cessation + 10 min (*p*’s ≤ 0.019). Moreover, separate analyses for both conditions revealed significant EP increases in reaction to both the TSST (main effect of time: *F*(1.46, 46.55) = 102.29, *p* < 0.001, η_p_^2^ = 0.76, *f* = 1.78) and the PlacTSST (main effect of time: *F*(2, 44) = 14.25, *p* < 0.001, η_p_^2^ = 0.39, *f* = 0.80). The EP levels were increased as compared to baseline at +1 min and + 10 min after TSST/PlacTSST cessation in both conditions (*p*’s ≤ 0.006).

Norepinephrine: We observed higher plasma NEP increases in the TSST participants as compared to the PlacTSST participants (interaction effect time-by-condition: *F*(2, 108) = 23.66, *p* < 0.001, η_p_^2^ = 0.31, *f* = 0.67). Controlling for potential confounders did not significantly change these results (interaction effects of time-by-condition: with age, BMI: *F*(2, 104) = 22.15, *p* < 0.001, η_p_^2^ = 0.30, *f* = 0.65; with age, BMI, TICS: *F*(2, 100) = 21.19, *p* < 0.001, η_p_^2^ = 0.30, *f* = 0.65). Post-hoc testing revealed significant differences in NEP levels between TSST participants and PlacTSST participants +1 min and + 10 min after TSST/PlacTSST cessation (*p*’s ≤ 0.032). Moreover, separate analyses for both conditions revealed significant NEP increases in reaction to both the TSST (main effect of time: *F*(2, 64) = 169.59, *p* < 0.001, η_p_^2^ = 0.84, *f* = 2.29) and the PlacTSST (main effect of time: *F*(2, 44) = 39.43, *p* < 0.001, η_p_^2^ = 0.64, *f* = 1.33). TSST participants exhibited increased NEP levels as compared to baseline +1 min and + 10 min after TSST cessation (*p*’s ≤ 0.002), whereas, in PlacTSST participants, NEP levels were (marginal) significantly increased +1 min and +10 min after PlacTSST cessation (*p*’s ≤ 0.078).

ACTH: The TSST participants exhibited higher plasma ACTH increases in reaction to the TSST as compared to the PlacTSST participants in reaction to the PlacTSST (interaction effect time-by-condition: *F*(1.85, 99.79) = 6.41, *p* = 0.003, η_p_^2^ = 0.11, *f* = 0.35). The effect was independent of potential confounders (interaction effects of time-by-condition: with age, BMI: *F*(1.92, 99.70) = 6.41, *p* = 0.003, η_p_^2^ = 0.11 *f* = 0.35; with age, BMI, TICS: *F*(1.96, 98.08) = 5.97, *p* = 0.004, η_p_^2^ = 0.11, *f* = 0.35). Post-hoc testing revealed significant differences between the TSST participants and PlacTSST participants at all post-TSST/-PlacTSST measurement timepoints (*p*’s ≤ 0.005). Separate analyses for both conditions revealed significant increases in reaction to both the TSST (main effect of time: *F*(1.74, 59.31) = 26.71, *p* < 0.001, η_p_^2^ = 0.44, *f* = 0.89) and the PlacTSST (main effect of time: *F*(3, 60) = 5.17, *p* = 0.006, η_p_^2^ = 0.21, *f* = 0.52). While the TSST participants exhibited elevated ACTH levels as compared to baseline at all post-TSST measurement timepoints (*p*’s ≤ 0.001), (marginal) significantly elevated ACTH levels as compared to baseline were observed for PlacTSST participants +1 and +10 min after PlacTSST cessation (*p*’s ≤ 0.050), although these returned to baseline levels at +20 min (*p* = 0.50).

Cortisol: The TSST induced significantly higher increases in salivary CORT in the TSST participants as compared to the PlacTSST in PlacTSST participants (interaction effect time-by-condition: *F*(2.04, 114.15) = 24.68, *p* < 0.001, η_p_^2^ = 0.31, *f* = 0.67). This effect was independent of confounders (interaction effects of time-by-condition: with age, BMI: *F*(2.22, 119.70) = 23.15, *p* < 0.001, η_p_^2^ = 0.30, *f* = 0.65; with age, BMI, TICS: *F*(2.41, 123.00) = 20.36, *p* < 0.001, η_p_^2^ = 0.29, *f* = 0.64). Post-hoc testing revealed significant differences between TSST participants and PlacTSST participants at all post-TSST/PlacTSST measurement timepoints (*p*’s ≤ 0.001). Moreover, analyses for conditions separately revealed that the TSST participants showed significant increases in CORT (main effect of time: *F*(1.74, 61.00) = 35.86, *p* < 0.001, η_p_^2^ = 0.51, *f* = 1.02), with elevated CORT levels as compared to baseline at all post-TSST measurement timepoints (*p*’s ≤ 0.001), whereas we observed a decrease in the PlacTSST participants (main effect of time: *F*((2.64, 55.33) = 11.01, *p* < 0.001, η_p_^2^ = 0.34, *f* = 0.72), with decreased CORT levels as compared to baseline +20 and +30 min after PlacTSST cessation (*p*’s ≤ 0.012).

Renin: We observed higher plasma REN increases in the TSST participants as compared to the PlacTSST participants (interaction effect time-by-condition: *F*(1.93, 109.70) = 4.67, *p* = 0.012, η_p_^2^ = 0.08, *f* = 0.29). Controlling for potential confounders did not significantly change these results (interaction effects of time-by-condition: with age, BMI: *F*(1.98, 108.71) = 4.23, *p* = 0.017, η_p_^2^ = 0.07, *f* = 0.27; with age, BMI, TICS: *F*(1.96, 99.98) = 3.70, *p* = 0.029, η_p_^2^ = 0.07, *f* = 0.27). There were significant differences between conditions +1 min after TSST/PlacTSST cessation (*p* = 0.046) but not at later measurement timepoints (*p*’s ≥ 0.48). Separate analyses for both conditions revealed significant increases in reaction to both the TSST (main effect of time: *F*(1.78, 60.52) = 18.73, *p* < 0.001, η_p_^2^ = 0.36, *f* = 0.75) and the PlacTSST (main effect of time: *F*(2.95, 78.78) = 11.11, *p* < 0.001, η_p_^2^ = 0.33, *f* = 0.70). Further post-hoc testing for TSST participants only revealed that, as compared to baseline, (marginal) significantly increased REN concentrations were observed +1 and +10 min after TSST cessation (*p*’s ≤ 0.089) but not at later measurement timepoints (*p*’s ≥ 0.085). For the PlacTSST participants, REN levels were elevated as compared to baseline at the first two measurement timepoints after PlacTSST cessation (*p*’s ≤ 0.003) but not later (*p*’s ≥ 0.31).

Aldosterone: The TSST induced (marginally) significant higher plasma ALD increases as compared to the PlacTSST (interaction effects time-by-condition: *F*(1.94, 114.66) = 3.16, *p* = 0.048, η_p_^2^ = 0.05, *f* = 0.23; with age, BMI: *F*(2.01, 114.58) = 2.77, *p* = 0.067, η_p_^2^ = 0.05, *f* = 0.23; with age, BMI, TICS: *F*(2.02, 107.22) = 2.37, *p* = 0.098; η_p_^2^ = 0.04, *f* = 0.20). Post-hoc testing revealed significant differences between TSST participants and PlacTSST participants +1, +10, and +20 min after TSST/PlacTSST cessation (*p*’s ≤ 0.048) but not +30 min after treatment cessation (*p* = 0.13). Moreover, separate analyses for both conditions revealed significant ALD increases in reaction to both the TSST (main effect of time: *F*(1.69, 59.02) = 86.82, *p* < 0.001, η_p_^2^ = 0.71, *f* = 1.56) and the PlacTSST (main effect of time: *F*(2.07, 49.61) = 22.68, *p* < 0.001, η_p_^2^ = 0.49, *f* = 0.98). Notably, for both conditions, we observed elevated ALD levels as compared to baseline at all post-TSST/-PlacTSST measurement timepoints (*p*’s ≤ 0.001).

#### Appendix A.1.3. Mediation Analyses

Higher psychosocial stress-induced increases in neutrophil granulocyte counts were only observed in analyses without correction for stress hemoconcentration effects. We therefore additionally conducted mediation analyses, testing for mediation effects of endocrine parameters and/or BV changes on neutrophil granulocyte count increases in reaction to acute psychosocial stress and subsequent decreases.

Mediation of neutrophil granulocyte count increases. First, we performed exploratory mediation analyses with the condition (TSST vs. PlacTSST) as the predictor, increases in neutrophil granulocyte counts as the criterion, and changes in BV, EP, NEP, ACTH, CORT, REN, or ALD from baseline to peak (+1 min) entered separately as potential mediators. Significant indirect effects were found for (1) BV change (ab = −0.27; 95% CI [−0.59; −0.05]) and (2) EP increase (ab = −0.36; 95% CI [−0.68; −0.17]). There were no significant indirect effects for NEP (95% CI [−0.38; 0.41]), ACTH (95% CI [−0.44; 0.65]), CORT (95% CI [−0.33; 0.54]), REN (95% CI [−0.017; 0.19]), or ALD (95% CI [−0.06; 0.30]).

Second, in the main mediation analyses, we entered all identified potential mediators of the exploratory analyses simultaneously. EP increase was the only mediator that remained significant for neutrophil granulocyte counts (EP: ab = −0.32; 95% CI [−0.67; −0.08]; BV: 95% CI [−0.48; 0.16]). Additional controlling for age and BMI did not significantly change these results (EP: ab = −0.33; 95% CI [−71; −0.09]; BV: 95% CI [−0.46; 0.22]).

Mediation of neutrophil granulocyte count decreases: First, we again performed exploratory mediation analyses with the condition (TSST vs. PlacTSST) as the predictor, neutrophil granulocyte count decreases from +1 min to +10 min after TSST/PlacTSST cessation as criterion, and changes in BV, EP, NEP, ACTH, CORT, REN, or ALD from +1 min to +10 min after TSST/PlacTSST cessation entered separately as potential mediators. Significant indirect effects were found for (1) BV change (ab = 0.44; 95% CI [0.16; 0.75]), (2) EP change (ab = 0.51; 95% CI [0.22; 0.74]), (3) NEP change (ab = 0.29; 95% CI [0.02; 0.57]), (4) CORT change (ab = 0.27; 95% CI [0.03; 0.63]), and (6) REN change (ab = 0.19; 95% CI [0.06; 0.38]). There were no significant indirect effects for ACTH change (95% CI [−0.27; 0.27]) and ALD change (95% CI [−0.06; 0.13]).

Second, in the main analyses, we entered all identified potential mediators of the exploratory analyses simultaneously. With respect to neutrophil granulocyte count decreases, changes in BV, EP and REN remained as significant mediators but none of the other exploratorily identified parameters, independent of age and BMI (BV: ab = 0.36; 95% CI [0.05; 0.81]; EP: ab = 0.40; 95% CI [0.04; 0.67]; REN: ab = 0.18; 95% CI [0.04; 0.36]; NEP: 95% CI [−0.32; 0.37]; CORT: 95% CI [−0.39; 0.18]; with age + BMI: BV: ab = 0.38; 95% CI [0.08; 0.82]; EP: ab = 0.39; 95% CI [0.01; 0.68]; REN: ab = 0.18; 95% CI [0.04; 0.38]; NE: 95% CI [−0.45; 0.40]; CORT: 95% CI [−0.36; 0.24]).
